# Differences in serum protein electrophoretic pattern in dogs naturally infected with *Babesia gibsoni* and *Babesia canis*

**DOI:** 10.1038/s41598-020-75908-7

**Published:** 2020-11-03

**Authors:** Csilla Tóthová, Martina Karasová, Lucia Blaňarová, Mária Fialkovičová, Oskar Nagy

**Affiliations:** 1 Clinic of Ruminants, University of Veterinary Medicine and Pharmacy, Komenského 73, 041 81 Kosice, Slovak Republic; 2 Clinic of Small Animals, University of Veterinary Medicine and Pharmacy, Komenského 73, 041 81 Kosice, Slovak Republic; 3 Parasitological Institute, Slovak Academy of Sciences, Hlinkova 3, 041 81 Kosice, Slovak Republic

**Keywords:** Diseases, Biochemistry, Proteins

## Abstract

Canine babesiosis may cause several hematological and biochemical changes, but only limited studies are available regarding the possible differences of changes in animals infected by different *Babesia* parasites. The study focused on the evaluation of the differences in serum protein electrophoretic pattern between dogs naturally infected with *B. gibsoni* (17 dogs) and *B. canis* (40 dogs). The mean values of total proteins, β_1_-, β_2_- and γ-globulins were in dogs infected with *B. gibsoni* significantly higher (*P* < 0.05 and *P* < 0.001) than in dogs infected with *B. canis*. The relative concentrations of albumin, α_1_-, α_2_-globulins and the A/G ratios were in the *B. gibsoni* infected dogs significantly lower (*P* < 0.001), no significant differences were found in the relative concentrations of β_1_- and β_2_-globulins. Significant differences were found in most of the evaluated parameters when comparing the results in relation to the form of *B. canis* infection to *B. gibsoni* infection. Hematological indices showed significant differences between dogs infected with *B. gibsoni* and the complicated form of *B. canis* infection. In conclusion, the obtained results suggest differences in the changes of serum protein electrophoretic pattern between dogs infected with both *Babesia* species and thus, in the response to the infection caused by various *Babesia* parasites.

## Introduction

Canine babesiosis is an important widespread haemoprotozoan parasitic infection documented principally throughout Asia, Africa, Japan and South America, but recently there has been an increase in the rate of transmission of such diseases which were previously considered as exotic or atypical for the dogs in Europe^1^. Infections have been reported also from various regions of Australia and North America^2^. The clinical manifestation, severity and the course of the disease primarily depend on the species of *Babesia* parasite causing infection^3^. While the *Babesia canis* (*B. canis*) infection is characterized by acute manifestation of clinical signs (marked hemolytic anaemia, hemoglobinuria, pale mucous membranes, icterus, consistent high fever), the clinical signs resulting from the infection with *Babesia gibsoni* (*B. gibsoni*) are less conspicuous than those observed in dogs infected with *B. canis*, and can vary widely from chronic or subclinical to fulminant disease resulting in multiple organ failure^4^. Hyper-acute states are rare, while chronic infections with lymph node enlargement, weight loss, protein-losing nephropathy, renal failure and immune-mediated hemolytic anemia are more common^5^. Dogs with chronic infections often do not show clinical signs of the disease (the so called carrier dogs), which is probably the result of the inability of the immune system to eliminate the infection^6,7^. Furthermore, PCR positive dogs without clinical signs of the disease and in the absence of microscopic parasitaemia have been also reported, probably caused by low parasitaemia below the microscopic detection limit and problematic detection of small-sized parasites in the blood smears^8^. Infections by *B. gibsoni* have been described predominantly in American pit bull terrier-type dogs in the absence of tick vectors, usually due to biting and fighting between infected and non-infected dogs, transmission by blood transfusions, as well as transplacental transmission^9,10^.

The pathophysiology and immunopathogenesis of the disease caused by *B. canis* is well described and documented in a large number of available reports, but the pathogenesis of *B. gibsoni* infection is incompletely understood. It was stated previously that the parasitic infection with *Babesia* induces a systemic inflammatory response and is associated with marked release of inflammatory cytokines^11,12^. In response to inflammatory cytokine secretion, increased production of acute phase proteins, particularly C-reactive protein were found in dogs naturally infected with *B. canis* at the time of presentation to the veterinary hospital, and recently also in dogs experimentally infected with *B. gibsoni*^13–15^. Furthermore, the infection with *B. canis* in dogs may cause marked alterations also in the electrophoretic pattern of serum proteins^16^, but the changes in the protein pattern in dogs infected with *B. gibsoni* remain undetermined. Many studies presented in the literature concerning babesiosis in dogs are focused on the issues of pathogenesis, clinical picture, some hematological and biochemical parameters, therapy and prevention. However, to our knowledge no studies have investigated the possible differences in the changes in haematological indices and serum protein profile in dogs suffering from babesiosis with regard to differences in the pathogenesis of various *Babesia* parasites. Therefore the study was focused on the evaluation of the alterations in the serum protein pattern in dogs naturally infected with *B. gibsoni*, and to compare the results with the values obtained in dogs infected with *B. canis* including also the comparison of uncomplicated and complicated forms of the disease. Because of the primary and main laboratory abnormalities in *Babesia* infections are hematological alterations, we evaluated the changes in some hematological parameters as well.

## Results

**Figure 1:**
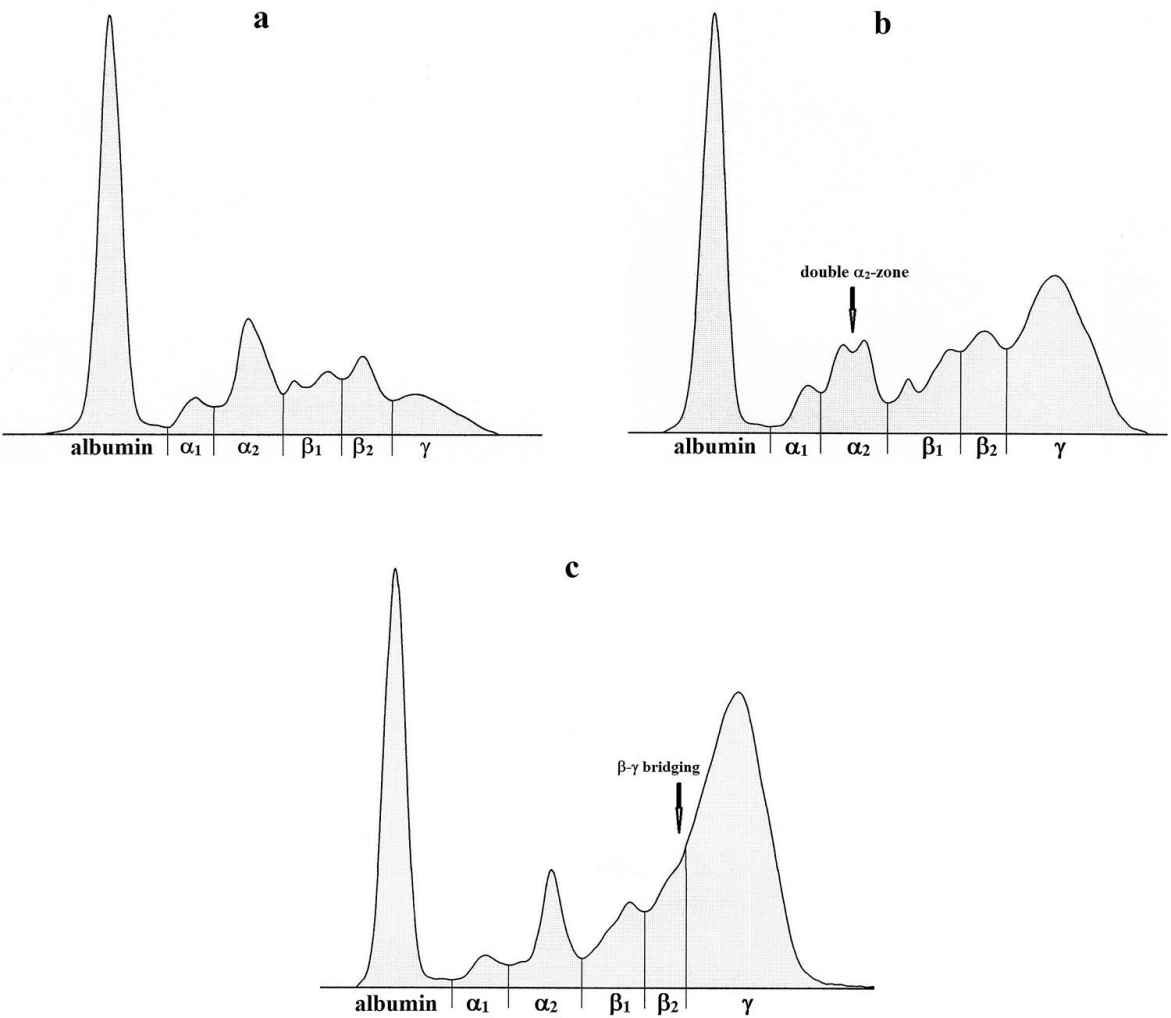
Representative electrophoretograms characterizing the differences in serum protein fractions in dogs infected with *B. canis* (**a**) and *B. gibsoni* (**b**,**c**).

The results of serum protein analyses and the hematological data are shown in Tables 1, 2 and 3. Representative examples of the electrophoretograms from both groups of dogs are shown in Fig. 1a–c.

**Table 1:** Differences in the relative concentrations of serum protein fractions (%) and albumin/globulin ratio (A/G) between dogs infected with *B. gibsoni* and *B. canis* (mean ± SD).

Variables	Dogs infected with				*P* value
	*B. gibsoni*	*B. canis*	*B. canis*		
			UC	C	
Albumin	34.9 ± 6.6	44.8 ± 6.2*	46.9 ± 5.5^c^	45.5 ± 5.8	< 0.001
α_1_-Globulins	3.5 ± 0.6	5.1 ± 1.2*	4.8 ± 0.9^c^	5.7 ± 1.3^c^	< 0.001
α_2_-Globulins	11.5 ± 1.8	16.6 ± 4.0*	16.8 ± 4.0^c^	16.3 ± 4.0^c^	< 0.001
β_1_-Globulins	11.2 ± 2.7	12.9 ± 4.1	10.9 ± 3.0	16.2 ± 3.7^b^	< 0.001
β_2_-Globulins	11.1 ± 2.0	11.1 ± 2.7	11.3 ± 2.9	10.9 ± 2.3	n.s
γ-Globulins	27.8 ± 5.9	9.4 ± 2.5*	9.5 ± 2.9^c^	9.4 ± 1.7^c^	< 0.001
A/G	0.55 ± 0.16	0.83 ± 0.21*	0.90 ± 0.19^c^	0.72 ± 0.19	< 0.001

*P* value—significance of the analysis of variance, n.s., not significant; A/G, albumin/globulin ratio; UC, uncomplicated form; C, complicated form.

*Significance of the differences between *B. gibsoni* and *B. canis* infected dogs at *P* < 0.001.

^b,c^Significance of the differences between *B. gibsoni* and *B. canis* UC and C infected dogs: ^b^*P* < 0.01; ^c^*P* < 0.001.

**Table 2:** Differences in the concentrations of total serum proteins (TP, g/l) and absolute values of protein fractions (g/l) between dogs infected with *B. gibsoni* and *B. canis* (mean ± SD).

Variables	Dogs infected with				*P* value
	*B. gibsoni*	*B. canis*	*B. canis*		
			UC	C	
TP	77.2 ± 6.7	56.2 ± 7.6^†^	58.0 ± 7.2^c^	53.1 ± 7.6^c^	< 0.001
Albumin	26.7 ± 3.9	25.3 ± 5.1	27.1 ± 4.3	22.1 ± 5.0^a^	< 0.01
α_1_-Globulins	2.7 ± 0.4	2.8 ± 0.5	2.7 ± 0.4	3.0 ± 0.5	n.s
α_2_-Globulins	8.8 ± 1.4	9.4 ± 2.7	9.8 ± 2.7	8.7 ± 2.5	n.s
β_1_-Globulins	8.7 ± 2.6	7.2 ± 2.3*	6.3 ± 1.9^b^	8.6 ± 2.4	< 0.01
β_2_-Globulins	8.6 ± 1.7	6.3 ± 1.8^†^	6.6 ± 2.0^b^	5.7 ± 1.2^c^	< 0.001
γ-Globulins	21.7 ± 6.3	5.3 ± 1.6^†^	5.5 ± 1.8^c^	5.0 ± 1.2	< 0.001

*P* value—significance of the analysis of variance, n.s., not significant; TP, total proteins; UC, uncomplicated form; C, complicated form.

*^,†^Significance of differences between *B. gibsoni* and *B. canis* infected dogs: *P < 0.05, ^†^*P* < 001.

^a,b,c^Significance of the differences between *B. gibsoni* and *B. canis* UC and C infected dogs: ^a^*P* < 0.05; ^b^*P* < 0.01;^c^*P* < 0.001.

**Table 3:** Differences in the values of hematological parameters between dogs infected with *B. gibsoni* and *B. canis* (mean ± SD).

Variables	Dogs infected with				*P* value
	*B. gibsoni*	*B. canis*	*B. canis*		
			UC	C	
RBC	4.41 ± 1.41	5.27 ± 1.72	5.88 ± 1.13^b^	4.24 ± 2.06	< 0.001
Hb	9.71 ± 3.07	11.54 ± 3.88	13.07 ± 2.77^b^	8.97 ± 4.18	< 0.001
PCV	0.29 ± 0.09	0.32 ± 0.11	0.37 ± 0.08^a^	0.24 ± 0.12	< 0.001

*P* value—significance of the analysis of variance, n.s., not significant; RBC, red blood cells; Hb, hemoglobin; PCV, packed cell volume; UC, uncomplicated form; C, complicated form.

^a,b^Significance of the differences between *B. gibsoni* and *B. canis* UC and C infected dogs: ^a^*P* < 0.05; ^b^*P* < 0.01.

The relative mean concentration of albumin was significantly lower in the dogs infected with *B. gibsoni* (about 10%) when compared to the mean value obtained in the dogs infected with *B. canis* (Table 1; *P* < 0.001). Similarly, the dogs infected with *B. gibsoni* had significantly lower relative concentrations of α_1_- and α_2_-globulins (*P* < 0.001). A double α_2_-zone was found in nine out of seventeen with *B. gibsoni* infected dogs (more than 50%) and in six out of forty dogs (15%) infected with *B. canis* (Fig. 1b). Non-significantly lower mean relative value of β_1_-globulins was found in dogs infected with *B. gibsoni*, the mean relative concentrations of β_2_-globulins showed no significant differences between the two groups of dogs and the mean values were similar. The dogs infected with *B. gibsoni* were found to have significantly higher relative mean values of γ-globulins (approximately threefold higher) than those infected with *B. canis* (*P* < 0.001). The γ-globulin pattern were in the dogs infected with *B. gibsoni* characterized by markedly higher values observable as higher and broad peaks on the electrophoretogram. Furthermore, a β–γ bridging with no clear separation of these fractions was observable in two from seventeen dogs infected with *B. gibsoni* (Fig. 1c). The A/G ratio was significantly lower in the group of dogs infected with *B. gibsoni* than in animals infected with *B. canis* (*P* < 0.001). Differences found in the results of the relative concentrations of the evaluated variables between the groups of dogs were significant (*P* < 0.001), except for β_2_-globulins. Compared to dogs with *B. gibsoni* infection, the mean relative values of albumin, α_1_-, α_2_-globulins and A/G ratio were significantly higher (*P* < 0.001) in dogs with uncomplicated form of *B. canis* infection. In dogs with complicated form of infection significantly higher mean relative values were found in α_1_- (*P* < 0.001), α_2_- (*P* < 0.001) and β_1_-globulins (*P* < 0.01). In both forms of *B. canis* infections the relative mean values of γ-globulins were significantly lower (*P* < 0.001) than in dogs with *B. gibsoni* infection.

Significantly higher mean total serum protein concentration was recorded in dogs infected with *B. gibsoni* when compared with the *B. canis* infected animals (*P* < 0.001) and this difference is more than 20 g/l (Table 2). The analyses of the absolute concentrations of protein fractions showed no significant differences in the mean albumin, α_1_- and α_2_-globulins between the two groups of dogs. Significantly higher mean values in dogs infected with *B. gibsoni* were observed for β_1_-, β_2_- and γ-globulins (*P* < 0.05, *P* < 0.001 and *P* < 0.001, respectively). The difference in the mean concentration of γ-globulins between both groups of dogs was more than 16 g/l. Except for α_1_- and α_2_-globulins the differences found in the means of the absolute concentrations of TP and protein fractions between dogs infected with *B. gibsoni* and the two forms of *B. canis* infections were significant (*P* < 0.01 and *P* < 0.001). The means of TP and absolute values of β_1_-, β_2_- and γ-globulins were significantly lower in dogs with uncomplicated form of *B. canis* infection than in dogs with *B. gibsoni* infection (*P* < 0.01 and *P* < 0.001). In dogs with complicated form of infection significantly lower means were found in TP (*P* < 0.001), albumin (*P* < 0.05) and β_2_-globulins (*P* < 0.001).

The evaluation of hematological parameters showed differences between the groups of dogs characterized by non-significantly lower mean number of red blood cells, lower mean concentration of hemoglobin and lower mean PCV value in dogs infected with *B. gibsoni* (Table 3). Differences found in the results of the relative concentrations of the hematological variables between dogs infected with *B. gibsoni* and the two forms of *B. canis* infections were significant (*P* < 0.001). The differences in the means of RBC, Hb and PCV were significant between the dogs infected with *B. gibsoni* and the complicated form of *B. canis* infection (*P* < 0.01, *P* < 0.01 and *P* < 0.05, respectively).

## Discussion

Babesiosis is one of the most important globally extended infections of dogs. Infections caused with *B. canis* are more common, but recently, due to global climate changes, *B. gibsoni* has been recognized as an important pathogen that affects dogs also in Middle Europe. Cases have been reported in Germany, Croatia, as well as from Serbia and Poland^1,17,18^. The infection with *B. gibsoni* mostly causes mild clinical signs which are manifested as a subclinical infection or associated only with weight loss and weakness^7^. Furthermore, chronic infections may be also observed that usually are completely asymptomatic or may be characterized by intermittent fever, lethargy, and weight loss^19,20^. In some cases, after initial parasitemia, the immune system may not totally eradicate the infection, and a chronic carrier state remains without clinical signs of the disease^21^. Relapses may occur months to years later and many complications may develop, including glomerulonephritis and polyarthritis^22^.

Hemolytic anemia is the predominant manifestation of babesiosis^23^. A significant decrease of total erythrocyte count and hemoglobin was reported in canine babesiosis by several authors indicating anemia in the affected dogs^24–26^. Although in dogs infected with *B. gibsoni* the parasitemia is usually mild, but anemia can be severe^21^. In earlier studies, hemoglobinuria with a severe hemolytic anemia was not observed in dogs naturally or experimentally infected with *B. gibsoni*^27,28^. In a further study, dogs experimentally infected with *B. gibsoni* developed mild anemia, which was transient and resolved by the day 17 after the infection^15^. On the other hand, Ishimine et al.^4^ observed severe hemolytic anemia in the peripheral blood of beagles infected with *B. gibsoni* on the 2nd to the 3rd week post infection, induced by the destruction of red blood cells by parasites as they leave red blood cells. Similarly, a significant reduction in RBC and hemoglobin concentration was recorded by Bilwal et al.^29^, Adaszek et al.^1^ and Yogeshpriya et al.^30^ in dogs naturally infected with *B. gibsoni* when compared to apparently healthy dogs, as a result of antibody-mediated cytotoxic or non-immune mediated destruction of circulating red blood cells. In the present study, differences were obtained also between the two evaluated *Babesia* species, with markedly lower RBC values, Hb concentrations, as well as Hct values in dogs infected with *B. gibsoni* than in those with uncomplicated form of *B. canis* infection, suggesting a longer lasting process of red blood cell destruction by small *B. gibsoni* parasites. The complicated form of *B. canis* disease showed insignificant differences in the mean values of hematological parameters with lower values than in *B. gibsoni* infection.

The infection with *B. gibsoni* in dogs may cause several biochemical changes, but only limited studies are available on the analysis of pathological changes expressed as alterations in the protein pattern of the host to this infection, as well as on the possible differences between animals infected by different *Babesia* parasites. Bilwal et al.^29^ reported no significant differences in the values of total serum proteins between dogs with babesiosis and healthy dogs. Reddy et al.^31^ found in dogs infected with large *Babesia* parasites a significant reduction in total serum protein concentrations. On the other hand, Yogeshpriya et al.^30^ obtained in dogs naturally infected with *B. gibsoni* about 10 g/l higher total serum protein values than in healthy dogs, but these differences were not significant. Presented study showed in dogs infected with *B. gibsoni* significantly higher total serum protein concentrations (about 20 g/l higher) when compared to both forms of *B. canis* infected animals. These very high total serum protein values in dogs infected with *B. gibsoni* might be caused by markedly increased synthesis of globulins and probably reflect the response of the organism to severe chronic inflammation^32^, which is much more characteristic for the infection with *B. gibsoni*.

In general, significantly decreased concentrations of albumin were found by Yadav et al.^33^ and Vijayalakshmi et al.^34^ in dogs with babesiosis than in healthy dogs. Yogeshpriya et al.^30^ observed no marked differences in the albumin concentrations between dogs naturally infected with *B. gibsoni* and healthy dogs. On the other hand, the laboratory studies conducted by Adaszek et al.^1^ showed in dogs infected with *B. gibsoni* decreased concentrations of albumin. Even though the results of our study showed no marked differences in the absolute values of albumin between the dogs infected with *B. gibsoni* and all *B. canis* infected dogs, significantly lower mean value was recorded in the group of complicated form of *B. canis* infection compared to *B. gibsoni* group of dogs. The mean relative concentration of this protein fraction was significantly lower in the dogs infected with *B. gibsoni* (about 10% lower). This pattern was caused by significantly higher total serum protein concentrations in the *B. gibsoni* infected dogs. From these higher values calculated absolute concentrations of albumin showed in general no differences between the dogs infected with different *Babesia* parasites. The aforementioned lower relative concentrations of albumin in the *B. gibsoni* infected dogs might by related to glomerulonephritis and renal impairment due to the damage of renal cells by inflammatory mediators in the affected dogs, and consequent protein-losing nephropathy with glomerular leakage of proteins^22,35^. Furthermore, albumin has been termed as the major negative acute phase protein with markedly reduced synthesis during the acute phase response, seeing that the majority of amino acids are used mainly for the synthesis of positive acute phase proteins during the systemic inflammatory response caused by the infection^12,36^. The very low relative albumin concentrations in dogs infected with *B. gibsoni* suggest severe inflammatory processes with possible serious alterations in the affected dogs (despite no serious abnormalities and clinical signs suggestive of the infection).

The alterations in the serum protein electrophoretic pattern in dogs following natural infection with *B. canis* were studied previously by Maegraith et al.^37^ and Tella and Maegraith^38^, and recently by Lobetti et al.^39^ and Tóthová et al.^16^, while a decrease in the relative concentrations of albumin was a typical finding in these studies. Furthermore, Tóthová et al.^16^ recorded significantly higher relative concentrations of α_1_-, β_1_- and β_2_-globulins, and non-significantly higher values of α_2_- and γ-globulins in the affected dogs with a double α_2_-zone in six from thirty-seven evaluated animals. On the other hand, the findings of the study conducted by Lobetti et al.^39^ showed in dogs with mild and severe babesiosis low total serum proteins, albumin, A/G ratio and α-globulins, whereas dogs with complicated babesiosis had no typical alterations in the serum protein pattern. Changes in the serum protein pattern were observed also in dogs with *Babesia annae* infection, with differences depending on the presence or absence of azotaemia^40^. While the infected dogs without azotaemia had significantly higher total proteins and all the globulin fractions, and significantly lower albumin concentrations, the infected dogs with azotaemia showed significantly higher α_1_- and α_2_-globulins, and significantly lower albumin values compared to the non-infected dogs. On the other hand, the changes in the serum protein pattern associated with *B. gibsoni* infections in dogs are still poorly understood, including the possible differences between *B. gibsoni* and *B. canis* infected dogs. In this study presented results showed in the dogs infected with *B. gibsoni* significantly lower relative concentrations of α_1_- and α_2_-globulins when compared to *B. canis* infected dogs. The infection with *Babesia* parasites is associated with the development of systemic inflammatory responses, including the excessive release of inflammatory mediators and consequent increased production of acute phase proteins^12^. The results of the studies conducted by Matijatko et al.^14^ and Barić Rafaj et al.^41^ indicated that the natural infection with *B. canis* induced a marked acute phase response, characterized by higher concentrations of serum amyloid A that might be useful also in the monitoring of the response to treatment. Furthermore, some other proteins associated with the activation of host immune response were observed in *B. canis* infected dogs, including the increased synthesis of haptoglobin and ceruloplasmin^13^. The aforementioned acute phase proteins belong to the α-globulin fractions^42^, thus, the markedly higher concentrations of α_1_- and α_2_-globulins obtained in the dogs infected with *B. canis* may reflect the increased production of acute phase proteins from these fractions. When compared to the *B. canis* infected dogs, the animals infected with *B. gibsoni* had significantly lower relative concentrations of α_1_- and α_2_-globulins. Although an excessive proinflammatory activity was detected also in animals experimentally infected with *B. gibsoni*^15^, the increased synthesis of acute phase proteins from the α-fractions was not yet described. Seeing that infections with *B. gibsoni* are rather chronic and more prolonged, the tissue damage and inflammation in the infected dogs might not evoke sufficient inflammatory response to give a more marked increase in the concentrations of proteins from this fraction, and thus it may not be detectable on serum protein electrophoresis. On the other hand, lower concentrations of α-globulins in dogs with babesiosis may be caused by free hemoglobin due to intravascular haemolysis caused by the disease^43,44^. Furthermore, a double α_2_-zone was observable in our study in nine out of seventeen dogs infected with *B. gibsoni* (more than 50%) with no visible hemolysis in the samples, and in six out of forty dogs infected with *B. canis* (15%). Thus, this pattern of split α_2_-zone may suggest more severe intravascular haemolysis due to babesiosis or the presence of free hemoglobin (even in the absence of visible hemolysis). However, in the light of some contradictory data and due to missing or incomplete studies in the area of this research, further studies would be helpful to correctly explain these alterations. Some proteins from the α-fractions is difficult to separate electroforetically and identify due to their very low serum concentrations, therefore, the evaluation of individual serum proteins using other methods or immunoenzymatic assays are recommended.

In the relative concentrations of β-globulins, no significant differences were observed between the dogs infected with *B. gibsoni* and all dogs with *B. canis* infection, except for β_1_-globulin fraction in complicated form of *B. canis* infection. The values were higher than those obtained by Tóthová et al.^16^ in clinically healthy dogs. The infection with *Babesia* parasites activates the mechanisms of the non-specific immune responses of the host, including the complement pathway with increased synthesis of complement components, especially C3a^45,46^. Complement is involved also in the regulation of inflammatory processes^47^ and, thus, may be attributed to higher concentrations of β-globulins due to the infection with parasites and consequent tissue damage in the dogs with babesiosis when compared to healthy ones. C-reactive protein (CRP) is another protein that belongs to the β-globulin fraction. Based on the ability to react and the magnitude of its response during inflammatory processes, CRP was considered as the most sensitive and important positive acute phase protein in dogs with major diagnostic value^48,49^. Increased concentrations of CRP have been found in natural infection with *B. canis* caused by marked acute phase response^14,41^. It has been shown that CRP concentrations increase also in *B. rossi* and *B. gibson*i infections^13,15^. The higher absolute concentrations of β-globulins in *B. gibsoni* infected dogs, comparable with those observed in *B. canis* infections, suggest strong association between the components of this fraction and the increased synthesis of some acute phase proteins from these fractions, despite the chronic nature of infections with *B. gibsoni*. However, it should be taken into consideration that the concentrations of acute phase proteins, as well as serum protein fractions might be related to the stage of the disease at the time of sample collection.

In the study presented by Ishimine et al.^4^, the beta- and gamma-globulins increased remarkably in dogs experimentally infected with *B. gibsoni*, and the γ-globulins remained higher until the 24th week post infection. This increase was attributed to the humoral antibody responses against *B. gibsoni*, especially to the rapid increase of IgM and IgG immunoglobulins. Significantly increased concentrations of globulins in total were obtained also by Bilwal et al.^29^ in dogs naturally infected with *B. gibsoni*. When compared to dogs naturally infected with *B. canis*, presented study showed in dogs infected with *B. gibsoni* significantly higher relative concentrations of γ-globulins (approximately threefold higher). This pattern was characterized by higher and broad peaks in the γ-zone on the electrophoretogram, which might be attributed to the production of immunoglobulins (mainly IgG) directed against the invading agens and suggest chronic inflammatory processes and severe infection. Furthermore, as a consequence of antigenic stimulation by the *Babesia* parasites, the infected animals may produce also some other immunoglobulins, especially IgM or IgA in higher amounts^50^. These proteins may migrate into the β region or β–γ interzone and by overproduction may produce a beta–gamma fusion with no clear valley between these fractions^51^. This pattern, the so called β–γ bridging was observable in two out of seventeen dogs infected with *B. gibsoni*. The above mentioned changes in the concentrations of protein fractions resulted also in alterations in the A/G ratio. Decreased A/G ratio was found in dogs with babesiosis when compared to apparently healthy animals^29,33^. Differences were obtained also between the dogs infected with different *Babesia* parasites, with significantly lower A/G ratio in the animals infected with *B. gibsoni* than in *B. canis* infected dogs. The very low A/G ratios in the dogs infected with *B. gibsoni* were caused by decreased albumin concentrations and the overproduction of globulins.

## Conclusions

In conclusion, the results indicate that the serum protein electrophoretic pattern in dogs are significantly altered both by *B. gibsoni* and *B. canis* infections, with different nature and magnitude of changes in the infected dogs according to the species of *Babesia* parasite involved in the infection. The obtained results suggest marked differences in the response of the organism to the infection caused by different *Babesia* parasites. When compared to infections with *B. canis*, characteristic alterations in the serum protein associated with *B. gibsoni* infection in dogs include significantly higher total serum proteins and γ-globulins, while the relative concentrations of albumin and A/G ratio are markedly significantly lower. The results also suggest some differences when comparing the results of a group of dogs with *B. gibsoni* infection and evaluated forms of *B. canis* disease. Identification of those protein fractions whose serum concentrations undergo marked changes due to the infection with *B. gibsoni* may provide useful information for the understanding of metabolic and pathological processes occurring in the infected animals. Due to the increasing frequency of infections also in Europe, this study may be the first step towards further investigations of biochemical and metabolic changes in dogs infected with *B. gibsoni*.

## Material and methods

### Ethics declarations

This study was based on the standard clinical examination and blood sample collection. The blood samples were collected as per standard sampling procedure used without any harm to the animals. All procedures with animals in the study were conducted in accordance with the ethical standards and quidelines approved by the Committee of the University of Veterinary Medicine and Pharmacy in Košice on protection of animals used for scientific purposes and complied with the institutional requirements of the Code of Ethics for Scientists (Directive 74/2019/UVLF).

### Animals and sample collections

Blood samples from seventeen client-owned dogs naturally infected with *B. gibsoni* were selected for this study. The evaluated dogs were American pit bull terriers of both genders (9 males and 8 females), their ages ranged from 1 to 5 years. Only pale mucouse membranes were observed by clinical examination in the evaluated dogs, no other abnormalities or signs suggestive of the infection were documented, and vital parameters were found to be normal. The dogs were fed in the standard way and regularly subjected to prophylaxis against ecto- and endo-parasites, and vaccinations against primary infectious diseases.

To compare the results between the two types of *Babesia* infections, forty dogs naturally infected with *B. canis* were also included into the study. They were admitted to the Clinic of Small Animals of the University of Veterinary Medicine and Pharmacy in Košice (Slovak Republic) with various clinical signs consisted with *B. canis* infection. Based on clinical symptoms and results of hematological and biochemical examinations aimed at diagnosing the occurrence of complications in the course of the disease, the dogs were divided into a group with uncomplicated (group UC, 25 dogs) and complicated (group C, 15 dogs) form of *B. canis* infection. Dogs with uncomplicated form of babesiosis presented lethargy, anorexia, weakness, pale mucous membrane, icterus, hemoglobinuria and splenomegaly. Complicated cases of disease were associated with signs of systemic inflammatory response syndrome^52^ and multiple organ dysfunction syndrome^11^, and seven dogs of this group died. These dogs were of various breeds and both genders (29 males and 11 females) at the age of 6 months to 14 years.

Blood samples were collected for hematological and biochemical analyses, microscopic evaluation of blood smears and in case of *B. gibsoni* polymerase chain reaction (PCR). Samples from the *B. gibsoni* infected dogs were taken by the veterinarian directly at the dog breeding site. The dogs infected with *B. canis* were sampled at the clinic at the time of admission before treatment was started. Permission to complete blood samples was obtained from each dog owner. Blood was for hematological examination collected from *v. cephalica* into tubes with potassium ethylenediamine tetraacetic acid (EDTA) as anticoagulant (Sarstedt, Nümbrecht, Germany). Blood samples for the biochemical examinations, including protein analyses were taken into serum gel separator tubes without any additives or anticoagulants (Sarstedt, Nümbrecht, Germany). These samples were centrifuged within 2 h after collection at 3000*g* for 10 min and serum was transferred into plastic tubes. After the separation of sera, hemolysis was inspected. Hemolysis was present in 10 from 40 samples from dogs infected with *B. canis*. In samples from dogs infected with *B. gibsoni* no visible hemolysis was observed. One aliquot of the serum was dispensed into plastic tubes for protein analyses, and stored at − 20 °C until it was analyzed.

### Laboratory analyses

Diff-Quick stain (Medion Diagnostics AG, Düdingen, Switzerland) was used for the detection of *Babesia* organisms within red blood cells in the peripheral blood smears. The typical large pyriform parasites in the erythrocytes were identified as *B. canis*. The subspecies of *B. canis* were not detected. On the basis of the size of the intracellular parasites, the small forms of parasites observed in erythrocytes in blood smears were morphologically and morphometrically consistent with *B. gibsoni*, but the infection was confirmed by PCR using *B. gibsoni* specific primer.

Genomic DNA was extracted from 200 µL of EDTA-blood samples, using a commercial DNA extraction kit (GeneJET PCR Purification Kit, ThermoFischer Scientific, Lithuania). For the molecular detection of *Babesia* spp., PCR amplification of approximately 450 bp long fragment of 18S rRNA gene, spanned by a reverse BJ1 (5′GTCTTGTAATTGGAATGATGG3′) and forward BN2(5′TAGTTTATGGTTAGGACTACG3′) primer was performed according to Casati et al.^53^. Only positive samples were further screened for the presence of *Babesia gibsoni* by PCR using the primers Gib599F (5′CTCGGCTACTTGCCTTGTC3′) and Gib1270R (5′GCCGAAACTGAAATAACGGC3′), which targeted a 671-bp long fragment of the 18S rRNA gene^54^. In each PCR reaction, sequenced DNA from Babesia-positive dog was used as positive control and nucleases free water was added as the template in negative control. The PCR products were visualized by electrophoresis on 1.5% agarose gels stained with GoodView Nucleic Acid Stain (Beijing SBS Genetech, Beijing, China). All positive PCR products were purified using a purification kit (Qiagen, Hilden, Germany) and sequenced. Nucleotide sequences were manually edited in MEGA 6^55^ and further compared with GenBank entries by BLAST^56^. For the alignment of the homologous nucleotide sequences the Clustal W program was used.

Potassium-EDTA anticoagulated whole blood was used to assess the number of red blood cells (RBC, T/l), hemoglobin concentration (Hb, g/dl) and packed cell volume (PCV, %). Complete hematological analysis was done using the ProCyte Dx automated hematology analyzer (IDEXX Laboratories, Westbrook, Maine, USA). To evaluate the changes in the protein profile, serum samples were analyzed for the concentrations of total proteins and main protein fractions. The total protein concentrations (TP, g/l) were determined using an automated biochemical analyser Alizé (Lisabio, Poully en Auxois, France) according to the biuret method with commercially available diagnostic kits (Randox, Crumlin, United Kingdom). Zone electrophoresis on an agarose gel using an automated electrophoresis system Hydrasys and commercial diagnostic kits Hydragel 7 Proteine (Sebia Corporate, Lisses, Evry Cedex, France) was used for the separation of serum protein fractions^57^. The following protein fractions were identified: albumin, α_1_-, α_2_-, β_1_-, β_2_- and γ-globulins. They were expressed as relative values (%) according to the optical density and their absolute concentrations (g/l) were quantified from the TP concentrations. The ratios of albumin to globulins (A/G) were calculated also. The values obtained from dogs infected with *B. gibsoni* were compared to those infected with *B. canis*.

### Statistical analyses

The data obtained were subjected to statistical analysis. Descriptive statistical procedures were used to calculate arithmetic means (x) and standard deviations (SD) for each evaluated variable and group of dogs. The distribution of data was evaluated by Kolmogorov–Smirnov Test for normality. Not all the evaluated parameters showed normal distribution. Unpaired t-test was carried out to compare and evaluate the significance of differences in means between the *B. gibsoni* and *B. canis* infected dogs. The significance of differences in values between the *B. gibsoni* infected dogs and the uncomplicated and complicated forms of *B. canis* infections was evaluated using one-way ANOVA and Tukey–Kramer post-hoc test for normally distributed data and Kruskal–Wallis test with Dunn’s Multiple Comparisons post-hoc test for non-normally distributed data. Variables with *P* < 0.05 were considered as statistically significant. The statistical analyses were carried out by using the program GraphPad Prism V5.02 (GraphPad Software Inc., California, USA).

## Data Availability

The datasets generated and/or analysed during the current study are available from the corresponding author on reasonable request.
